# Snaring Self-Expanding Devices to Facilitate Transcatheter Aortic Valve Replacement in Patients with Complex Aortic Anatomies

**DOI:** 10.3390/jcm12155067

**Published:** 2023-08-01

**Authors:** Yi-Jun Yao, Zhen-Gang Zhao, Xi Wang, Yong Peng, Jia-Fu Wei, Sen He, Yi-Jian Li, Jing-Jing He, Zhong-Kai Zhu, Yuan Feng, Mao Chen

**Affiliations:** Department of Cardiology, West China Hospital, Sichuan University, Chengdu 610041, China; yijun_yao@foxmail.com (Y.-J.Y.); pengyongcd@126.com (Y.P.); weijiafu@163.com (J.-F.W.); happensky@163.com (S.H.); liyijian_first_email@foxmail.com (Y.-J.L.); h382508867@gmail.com (J.-J.H.); zhongkaizhu92@gmail.com (Z.-K.Z.)

**Keywords:** transcatheter aortic valve replacement, aortic stenosis, snare, annulus angulation, bicuspid aortic valve

## Abstract

The snare-assisted technique has been described to facilitate transcatheter aortic valve replacement (TAVR) delivery system advancement in complex aortic anatomies. However, the evidence is limited to case reports. To evaluate the safety profile of the snare-facilitated approach and its impact on self-expanding (SE) TAVR outcomes, we collected consecutive patients who underwent transfemoral SE-TAVR for aortic stenosis, using propensity score matching (PSM) separately in tricuspid and type-0 and type-1 bicuspid aortic valve morphology between the snare and non-snare groups. In 766 patients, despite the snare group having significantly larger annulus angulation and maximal ascending aortic diameter, both groups achieved comparable 30-day device success rates, regardless of first-generation or new-generation valve use. After PSM, the snare group had a significantly lower new permanent pacemaker implantation rate among 193 type-0 patients (3.3% vs. 18.3%, *p* = 0.01). The ipsilateral group used new-generation valves less frequently (23.0% vs. 75.4%, *p* < 0.001), but there were no significant inter-group differences in procedure-related events, except for a lower incidence of PVL ≥ mild in the ipsilateral group (14.9% vs. 32.3%, *p* = 0.01). In conclusion, the snare-assisted technique appears useful in SE-TAVR with angulated aortic root anatomy, and the benefits were comparable between ipsilateral and contralateral snare techniques.

## 1. Introduction

Transcatheter aortic valve replacement (TAVR) has matured as an established treatment for aortic stenosis with proven safety and efficacy in randomized trials involving patients of all surgical risk categories [1,2,3]. However, TAVR remains technically challenging in patients with complex aortic anatomies, including the severely calcified aortic valve, bicuspid aortic valve, horizontal aorta, and concomitant aortic aneurysm [4,5,6,7,8].

In such cases, the delivery of TAVR devices could be challenging, particularly when using self-expanding (SE) valves, and may even fail to cross the severely calcified aortic valve or catastrophic complications such as aortic dissection [6,7,8]. Although balloon-expandable (BE) valves have better deliverability owing to the shorter stent frame and deflectable delivery system, they have been associated with a higher risk for annular rupture in severe valvular, annular, or sub-annular calcification [9,10].

The dilemma has led to the application of special maneuvers to facilitate the delivery of SE-TAVR valves in complex anatomies mainly caused by the bicuspid aortic valve and horizontal aorta. Typically, the prosthesis is captured with a contralateral or ipsilateral snare catheter, on which an external traction force is applied to pull the delivery system toward the inner curvature to facilitate its advancement through the ascending aorta and aortic valve. However, reported experiences with the approach are limited to case reports or case series [11,12,13,14,15,16].

Our center has been employing the snare-facilitated technique in a systematic manner for patients with complex aortic anatomies and expected difficulty in valve delivery. We aim to evaluate the safety profile of the snare-facilitated approach and its impact on SE-TAVR outcomes in patients with complex anatomies.

## 2. Materials and Methods

### 2.1. Study Population

**Figure 1:**
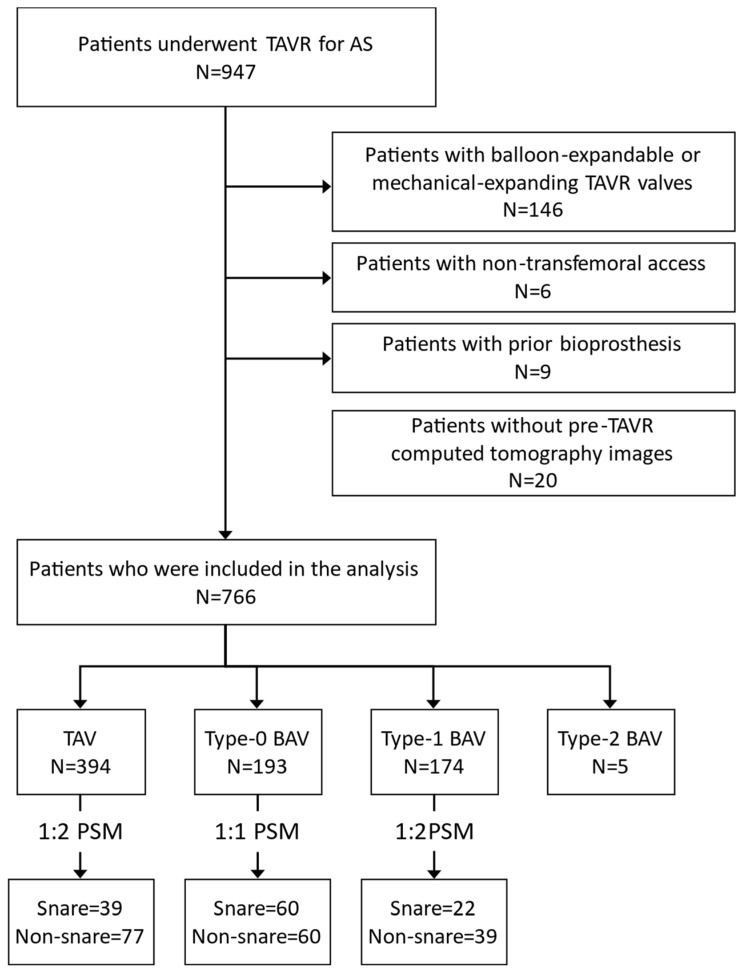
Study flow diagram. AS: aortic stenosis; BAV: bicuspid aortic valve; PSM: propensity score matching; TAV: tricuspid aortic valve; TAVR: transcatheter aortic valve replacement.

From January 2019 to December 2022, 947 patients underwent TAVR for severe aortic stenosis at West China Hospital, Sichuan University. After applying the exclusion criteria of balloon-expandable and mechanical-expanding TAVR valves, non-transfemoral access, prior bioprosthesis, and absence of pre-TAVR computed tomography (CT) images, 766 patients with severe native aortic stenosis were treated with transfemoral SE-TAVR and included in the analysis (Figure 1).

### 2.2. Computed Tomography Image Acquisition and Analysis

Electrocardiogram-gated multi-detector CT was performed prior to TAVR in all of the patients, using a second-generation dual-source CT scanner system (Siemens SOMATOM Definition Flash, Siemens Healthcare, Erlangen, Germany) or a new-generation Gemstone spectral imaging CT scanner system (GE Revolution, GE Healthcare, Chicago, IL, USA). About 80 mL of iodinated contrast agent was administered intravenously. Tube potential was set to 100 to 120 kV according to the patient’s body mass index, and tube current was adjusted based on individual patient’s size.

**Figure 2:**
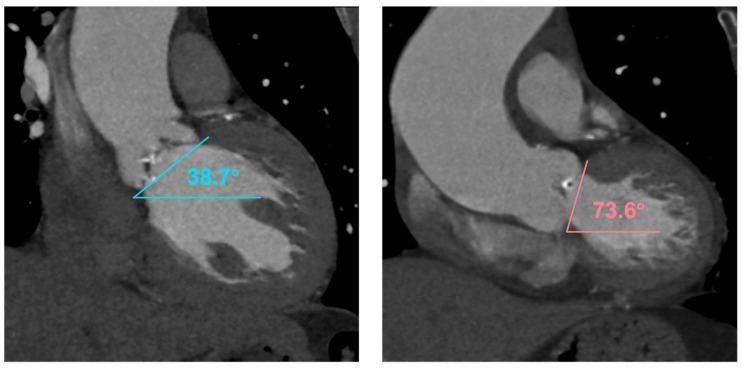
Measurement of annulus angulation on CT. Annulus angulation was measured as the angle between the horizontal plane and the annulus plane on coronal projection of reconstructed computed tomographic images. A representative image of non-horizontal and horizontal aorta was presented.

CT Digital Imaging and Communications in Medicine (DICOM) data were analyzed by trained physicians using the OsiriX Dicom Viewer software (OsiriX Foundation) and over-read by an expert CT imager (Y.F.). The aortic valve morphology was identified as tricuspid aortic valve (TAV) and type-0 and type-1 bicuspid aortic valve (BAV) according to Sievers’ classification [17]. In patients with BAV, both annular and supra-annular dimensions were measured for sizing purposes, as described previously [18]. The severity of aortic valve calcium was quantified using contrast scans with the threshold of detection set at 850 Hounsfield units (HU). Annulus angulation (AA) was measured as the angle between the aortic annular plane and the horizontal plane on coronal projection (Figure 2). Horizontal aorta was defined as an AA ≥ 50°, and extremely horizontal aorta was defined as an AA ≥ 70°.

### 2.3. Indications of Using Snare in SE-TAVR

The snare technique was used as a planned or bail-out procedure to change the direction of the nose-cone of the SE-TAVR delivery system and, thus, facilitate its advancement.

Planned use of snare was typically in cases where meticulous preprocedural MDCT analysis detected at least one of the following anatomical challenges implying difficulty or high complication risk in advancing the delivery system: (1) horizontal or extremely horizontal aorta; (2) significantly dilated ascending aorta (≥45 mm); (3) the presence of calcific or fibrotic commissural fusion between the right-coronary and non-coronary cusps.

Snare was used as a bail out in the following scenarios: (1) unexpected failure in delivering the SE-TAVR system across the stenotic aortic valve; (2) unexpected difficulty in exchanging catheters or delivering valvuloplasty balloon across the aortic valve; (3) unstable track (extra stiff guidewire) noted when attempting to cross the aortic valve.

Notably, the presence of an acute aortic arch (type III aortic arch) alone does not prompt the use of snare, which seems unlikely to facilitate the passage of the delivery system through the aortic arch due to the parallel relationship between the snare catheter and the delivery system at this stage.

### 2.4. Procedural Description

**Figure 3:**
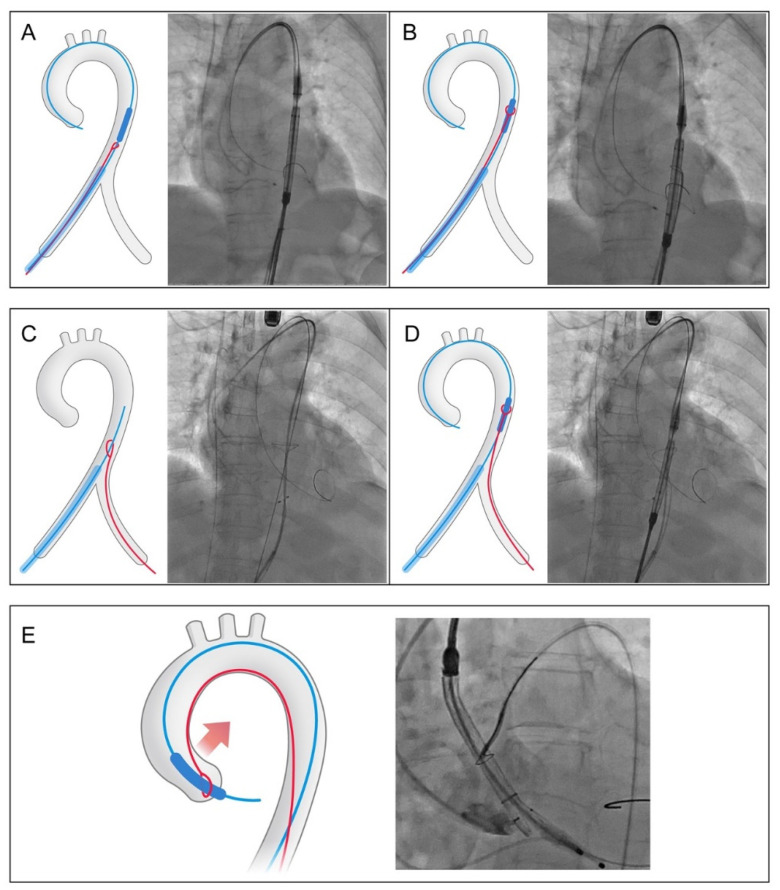
Procedural description and fluoroscopic images of ipsilateral and contralateral snare. (**A**) When using ipsilateral snare, the snare loop was pre-mounted on the delivery catheter just below the capsule and introduced simultaneously via the 20 Fr or 22 Fr GORE^®^ DrySeal Flex Introducer Sheath (WL GORE, Newark, DE, USA). (**B**) In the thoracic aorta, the snare loop was unfastened and advanced to hold the initial third of the capsule. (**C**) When using contralateral snare, the snare was placed in advance in the abdominal or thoracic aorta to catch hold of the workhorse guidewire (**D**) and the whole delivery system. (**E**) Further steps were similar between the ipsilateral and contralateral snare techniques. When the tip of the capsule arrives at the ascending aorta, the second operator applied an external traction force to pull the delivery system toward the inner curvature to facilitate its advancement through the ascending aorta and aortic valve.

The snare catheter was introduced through either the contralateral or ipsilateral femoral artery, with the latter being favored in our center. The detailed procedural description is shown in Figure 3 and Moving images S1–S5.

The ipsilateral snare technique is time-saving and more straightforward since it avoids manipulating the snare to capture the delivery system within the aorta. As an alternative, the contralateral snare could be used in patients with borderline femoral access (e.g., minimal lumen diameter < 6.0 mm) that mandates sheathless TAVR. In addition, the inline sheath design of some new-generation SE-TAVR systems is not compatible with ipsilateral snare.

The first-generation SE-TAVR systems that were used in our center included VenusA Valve (Venus MedTech, Hangzhou, China) and TaurusOne (Peijia Medical, Suzhou, China). The new-generation SE valves we used included Evolut R/Evolut PRO (Medtronic, Minneapolis, MN, USA), VenusA-plus Valve (Venus MedTech, Hangzhou, China), VitaFlow/VitaFlow II (MicroPort, Shanghai, China), TaurusElite/TaurusNXT (Peijia Medical, Suzhou, China), and Prostyle (KingstronBio, Suzhou, China).

As the default, and with no exception, all SE-TAVR procedures in our center were performed with the Lunderquist extra-stiff guidewire.

### 2.5. Outcome Assessment

The procedure-related events and short-term outcomes were reported according to the Valve Academic Research Consortium 3 recommendations. The severity of post-TAVR aortic regurgitation was qualitatively assessed and graded via transthoracic echocardiography according to established guidelines.

### 2.6. Statistical Analysis

Continuous variables were tested for normality of distribution using the Shapiro–Wilk test. Normally distributed continuous variables with normal distribution were presented as mean ± standard deviation (SD) and compared using Student’s *t*-test, while abnormally distributed continuous variables were presented as interquartile range and compared using the Mann–Whitney U test. Categorical variables were presented as numbers (percentage) and compared using chi-square or Fisher exact test.

Propensity score matching (PSM) was performed separately in different aortic valve morphology subgroups (TAV, type-0 BAV and type-1 BAV) between patients who underwent snare-assisted TAVR and those who did not. The concomitant baseline variables included age, mean aortic valve gradient, AA, maximal ascending aorta diameter, annular calcium size, and the use of new-generation valves. The groups were matched according to the propensity scores using nearest neighbor matching in a 1:2 or 1:1 ratio with a 0.1 caliper width and without replacement.

A two-tailed *p*-value < 0.05 was considered statistically significant. SPSS statistics software version 26.0 (IBM, Armonk, NY, USA) and R statistics software version 3.5.0 were used for data analysis.

## 3. Results

Among the 766 patients undergoing SE-TAVR, 394 (51.4%) had a TAV, and 372 (48.6%) had a BAV, including 193 with type-0 BAV, 174 with type-1 BAV, and 5 with type-2 BAV. The average AA was 53.5 ± 10.0° in the entire cohort. BAV patients had larger AA than TAV patients (54.2 ± 10.5° vs. 52.7 ± 9.8°, *p* = 0.04). There is a significant negative linear correlation between height and AA (per meter, β coefficient −18.83, 95% CI −31.34 to −6.32, *p* = 0.003). The frequency of adopting the snare-assisted technique was 18.1%, and it was higher in patients with type-2 BAV (60%) and type-0 BAV (35.8%) than in those with type-1 BAV (14.9%) and TAV (10.4%).

As shown in Table 1, the snare group had significantly larger AA (60.2 ± 10.1° vs. 52.0 ± 9.3°, *p* < 0.001), larger maximal ascending aorta diameter (42.6 ± 6.5 mm vs. 39.7 ± 5.1 mm, *p* < 0.001), larger STJ diameter (32.2 ± 4.6 mm vs. 30.0 ± 4.1 mm, *p* < 0.001), higher left coronary ostium height (14.3 ± 4.1 mm vs. 13.5 ± 3.3 mm, *p* = 0.03), smaller left ventricular dimension (LVEDD, 50 ± 8 mm vs. 53 ± 9 mm, *p* = 0.001), a lower proportion of pre-TAVR aortic regurgitation ≥ moderate (15.8% vs. 29.1%, *p* = 0.001), and a higher proportion of type-0 BAV anatomy (70.5% vs. 43.7%, *p* < 0.001) than the non-snare group. The proportion of using first-generation valves was lower in the snare group than in the non-snare group (52.5% vs. 69.1%, *p* < 0.001). The snare group achieved comparable 30-day device success rates and other procedure-related events to the non-snare group. Other baseline characteristics and procedural data in patients with first-generation and new-generation valves was shown in Table S1.

### 3.1. Outcomes after PSM According to Aortic Valve Morphology

Among type-0 BAV patients, due to the high frequency of snare usage in the entire cohort, a 1:1 PSM was performed. There were 60 patients in the snare group and 60 patients in the non-snare group were included for further analyses. As shown in Table S2, the baseline characteristics were well balanced between the two groups, including the AA (56.9 ± 10.7° vs. 55.0 ± 8.4°, *p*= 0.28), maximal ascending aorta diameter (43.7 ± 4.4 mm vs. 43.6 ± 4.9 mm, *p* = 0.96) and the usage of new-generation valves (40.0% vs. 31.7%, *p* = 0.34). There was no significant difference in procedure-related events, except for the notably lower rate of permanent pacemaker implantation in the snare group (3.3% vs. 18.3%, *p* = 0.01). This difference persisted among the patients treated with new-generation valves, with a significantly lower incidence of permanent pacemaker implantation in the snare group (0.0% vs. 26.3%, *p* = 0.01).

In patients with type-1 BAV, 22 in the snare group and 39 in the non-snare group were included after a 1:2 PSM, with the baseline characteristics well balanced between the two groups. There was a significantly lower frequency of post-dilation in the snare group (29.3% vs. 69.2%, *p* = 0.002), while the other procedure-related events showed no significant inter-group differences (Table S3).

In TAV patients, 39 in the snare group and 77 in the non-snare group were included after a 1:2 PSM. Despite the AA was still significantly larger in the snare group (56.7 ± 6.7° vs. 63.0 ± 8.1°, *p* < 0.001), there were no significant inter-group differences in the incidence of second valve implantation, new permanent pacemaker implantation, vascular complications, bleeding, cardiac tamponade, PVL ≥ mild, 30-day device success, 30-day stroke, or 30-day all-cause death (Table S4).

### 3.2. Ipsilateral Versus Contralateral Snare-Assisted Technique

Of the 139 patients undergoing snare-assisted SE-TAVR in the entire cohort, the snare was introduced from the contralateral and ipsilateral femoral arteries in 65 (46.8%) and 74 (53.2%), respectively (Table S5). The dimensions of the aortic root and the aorta were comparable between the two groups. New-generation valves were more frequently used in the contralateral group (75.4% vs. 23.0%, *p* < 0.001). There was a significantly lower incidence of PVL ≥ mild in the ipsilateral group (14.9% vs. 32.3%, *p* = 0.01), while the approach of the snare-assisted technique, ipsilateral or contralateral, did not seem to impact the procedure-related events, including the device success rate, cardiac tamponade, aortic dissection, vascular complications, and bleeding.

## 4. Discussion

We assessed the usefulness of snare for SE-TAVR in challenging anatomical settings with anticipated difficulty or failed attempts in direct valve delivery. The major findings could be summarized as follows: (1) snare-assisted SE-TAVR in highly challenging aortic anatomies (manifested as more horizontal and dilated aortas) achieved satisfactory procedural and short-term clinical results, which are comparable to those observed with standard TAVR in less challenging anatomies, regardless of using first-generation or new-generation SE-TAVR systems; (2) the results remained stable in different subgroups (type-0 BAV, type-1 BAV, and TAV); (3) the approach for introducing snare, ipsilateral or contralateral, exhibited similar safety profile and effectiveness.

The horizontal aorta represents an important anatomical challenge for TAVR, particularly with SE valves. Abramowitz et al. were among the first to systematically assess the impact of CT-determined AA on procedural results of TAVR [19]. They found that AA ≥ 48° was associated with a significantly lower procedural success rate in 102 patients undergoing TAVR with first-generation SE valves (CoreValve; 76.1% vs. 96.4%, *p* < 0.002). In contrast, increased AA did not influence acute procedural results among the 480 patients undergoing BE-TAVR. The different effects of AA on SE-TAVR versus BE-TAVR could be attributed to the relatively shorter stent frame and the deflectable delivery system of BE valves, which allow operators to more easily direct the system across the stenotic aortic valve, optimize coaxial valve alignment and thus achieve more predictable results in case of challenging aortic anatomy. When implanting SE valves, the delivery system of which is more rigid and not actively flexible, difficulties could be encountered when advancing the delivery system through the ascending aorta, crossing the aortic valve, and attempting coaxial deployment in the presence of the horizontal aorta. The coexistence of dilated aorta and severe aortic valve calcium usually increases complexity.

The new-generation SE valves, which are repositionable and recapturable, have been reported to produce improved TAVR outcomes. However, implanting new-generation SE valves in patients with increased AA remains challenging and associated with less favorable outcomes in some studies. Two studies [20,21] reported no influence of increased AA on procedural and clinical outcomes in 136 and 146 patients undergoing SE-TAVR with Evolut R. Another study analyzed 1903 patients who used the ACURATE valve, and there was no significant difference in device success rate between patients with AA ≥ 49° and those with AA < 49° (91.2% vs. 90.0%, *p* = 0.37) [7]. However, it was also reported that among the 1959 patients treated with Evolut R/PRO valves, the horizontal aorta was associated with a significantly lower device success rate (86.6% vs. 92.3%, *p* < 0.001), which remained consistent after adjusting for other outcome predictors (OR, 0.46 [95% CI, 0.31–0.70]) [7]. Indeed, the active flexion of the delivery system, an important feature to cope with the technical difficulty associated with the horizontal aorta, is still not possible with the currently available second-generation SE valves.

A few case reports have described the application of snare to overcome the difficulties in advancing the delivery system of SE valves [11,12,13,14,15]. However, there have been very limited data regarding the usefulness and safety of the maneuver. In this present study, we found that the incorporation of the snare in SE-TAVR facilitated the procedures and produced satisfactory acute and short-term results in challenging anatomies, comparable to those noted with standard SE-TAVR in less challenging anatomies. Importantly, the use of a snare did not bring additional risk of vascular complications. The main utility of the snare during SE-TAVR was to enable active flexion of the delivery system toward the inner curvature and, thus, facilitate its advancement through the ascending aorta and the aortic valve. In this way, excessive interaction between the delivery catheter, aorta, and aortic root could be reduced.

With the extension of TAVR into the relatively younger group, an increasing number of patients with BAV are being treated with TAVR [22]. However, TAVR in BAV remains challenging. One important technical difficulty is the commonly concomitant horizontal aorta, which could prevent the advancement of SE-TAVR across the stenotic aortic valve, particularly in instances of dilated ascending aorta and severe valve calcium [23,24]. In our study, patients with type-0 BAV more frequently required snare to assist with SE-valve delivery. In this subgroup, patients with more angulated and dilated aortas who underwent snare-assisted SE-TAVR had similar procedural results compared to those with less challenging anatomy and undergoing standard SE-TAVR. The data suggested that snare could serve as an effective tool for SE-TAVR in complex bicuspid anatomy. Interestingly, in the type-0 BAV subgroup, patients undergoing snare-assisted SE-TAVR had significantly less requirement for permanent pacemaker implantation (3.3% vs. 18.3%, *p* = 0.01). However, such a difference was not observed in the type-1 BAV and TAV subgroups. Therefore, the lower pacemaker implantation rate might be related more to the anatomical variables, such as different membranous septum length or supra-annular versus annular dimension, rather than the effect of the snare. Nevertheless, we do not exclude the possibility of less mechanical compression of the delivery catheter over the region of the conduction tissue as the result of the traction force by the snare as a contributor. Future dedicated studies are needed to unveil the underlying mechanisms.

According to the reported cases of snare-assisted SE-TAVR, the snare was usually introduced via the radial artery or contralateral femoral artery [11,12,13,14,15,16]. In our experience, however, the ipsilateral snare was the default technique, and in over 50% of the cases, the snare was introduced along with the delivery system via the same large sheath. The ipsilateral snare technique has the following advantages: (1) time-saving, as it exempts the need for additional arterial line and catheter manipulation for snaring the workhorse guidewire in advance; (2) safer, as it allows to preserve the workhorse guidewire (lifeline) in the left ventricle in bail-out use of snare. Importantly, according to our data, the ipsilateral snare technique exhibited a similar safety profile and effectiveness compared with the contralateral approach, despite the less use of the second-generation devices. Nevertheless, the ipsilateral snare is incompatible with sheathless SE-TAVR and is more demanding on the size of the iliofemoral artery. The selection of ipsilateral versus contralateral snare technique should be based on patients’ anatomy, type of device, and local institutional experiences.

Overall, it should be noted that the effect of the snare in SE-TAVR is primarily aligning the delivery system toward the center of the aorta and aortic root, thus facilitating its advancement. However, the coaxial implant remains challenging in the angulated aorta, as the snare should be of little help at the stage of valve deployment. A future iteration of a delivery system for SE valves enabling active flexion and extension, if technically achievable, would help operators to optimize coaxial valve advancement and alignment when faced with complex aortic anatomies.

The study findings should be interpreted in light of the following limitations. First, this study was non-randomized in design. Although PSM analyses based on key clinical, anatomical, and procedural variables were performed, some potential confounders might have been excluded from the matching process. Second, new-generation devices were used by around 30% of the patients in this study, which explained the relatively low overall rate of device success. Although the utility of snare in SE-TAVR is independent of the repositionability and recapturability of SE valves, we could not exclude the possible interaction of device type with the relationship between snare use and procedural outcomes. A future randomized study incorporating contemporary devices would help confirm the benefits of the snare-assisted technique in SE-TAVR.

## 5. Conclusions

The snare-assisted technique appears useful in SE-TAVR in challenging aortic anatomies featured primarily by the angulated aortic root to cope with anticipated difficulty or failure in valve delivery. The benefits were comparable between ipsilateral and contralateral snare techniques, suggesting that the ipsilateral snare technique could be a more time-efficient and less risky alternative to the contralateral approach for TAVI in certain situations.

## Figures and Tables

**Table 1:** Baseline characteristics and procedural data.

Variable	Non-Snare Group (*n* = 627)	Snare Group (*n* = 139)	*p*
Age, years	73 ± 8	73 ± 7	0.64
Male	352 (56.1)	76 (54.7)	0.76
BMI, kg/m^2^	22.9 ± 3.5	23.9 ± 3.8	0.002
Height, m	1.59 ± 0.08	1.59 ± 0.09	0.85
Hypertension	270 (43.1)	66 (47.5)	0.34
Diabetes	129 (20.6)	35 (25.2)	0.23
Cerebrovascular disease	95 (15.2)	20 (14.4)	0.82
Coronary artery disease	127 (20.3)	34 (24.5)	0.27
Atrial fibrillation	91 (14.5)	18 (12.9)	0.63
STS score, %	2.92 (1.94–4.76)	2.62 (1.91–4.15)	0.14
Echocardiographic data			
Ejection fraction, %	56 ± 15	59 ± 14	0.04
Mean aortic valve gradient, mmHg	56 ± 19	56 ± 20	0.85
Peak aortic valve velocity, m/s	4.7 ± 0.8	4.7 ± 0.8	0.85
Aortic regurgitation ≥ moderate	182 (29.1)	22 (15.8)	0.001
LVEDD, mm	53 ± 9	50 ± 8	0.001
CT data			
Annulus angulation, °	52.0 ± 9.3	60.2 ± 10.1	<0.001
Valve Type			<0.001
TAV	353 (56.3)	41 (29.5)	
Type-0 BAV	124 (19.8)	69 (49.6)	
Type-1 BAV	148 (23.6)	26 (18.7)	
Type-2 BAV	2 (0.3)	3 (2.2)	
Annular area, mm^2^	475.2 ± 258.3	460.3 ± 115.3	0.51
Annular perimeter, mm	78.2 ± 28.6	76.9 ± 9.5	0.59
Valve calcium volume, mm^3^	441 (190–791)	413 (120–809)	0.37
SOV perimeter, mm	108.9 ± 13.6	111.6 ± 14.1	0.04
STJ diameter, mm	30.0 ± 4.1	32.2 ± 4.6	<0.001
Left coronary ostium height, mm	13.5 ± 3.3	14.3 ± 4.1	0.03
Right coronary ostium height, mm	15.2 ± 3.7	15.5 ± 3.5	0.43
Maximal ascending aorta diameter, mm	39.7 ± 5.1	42.6 ± 6.5	<0.001
Procedural Data and Clinical Outcomes			
First-generation valve	433 (69.1)	73 (52.5)	<0.001
THV size more than 26 mm *	164 (26.2)	37 (26.6)	0.91
Post-dilation	303 (48.5)	62 (44.6)	0.41
Contrast volume, ml	306 ± 83	307 ± 77	0.93
Need for a second valve	44 (7)	14 (10.1)	0.22
Permanent pacemaker implantation	109 (17.4)	28 (20.1)	0.44
PVL ≥ mild	156 (24.9)	32 (23.2)	0.67
PVL ≥ moderate	4 (0.6)	1 (0.7)	0.63
Cardiac tamponade	2 (0.3)	1 (0.7)	0.45
Aortic dissection	4 (0.6)	1 (0.7)	0.63
Vascular complications			0.89
Minor	30 (4.8)	8 (5.8)	
Major	5 (0.8)	1 (0.7)	
Bleeding			0.72
Type 1	9 (1.4)	2 (1.4)	
Type 2	4 (0.6)	0 (0)	
Type 3	2 (0.3)	1 (0.7)	
Device success at 30 days			
Among TAV patients	297 (84.1)	33 (80.5)	0.55
Among Type-0 BAV patients	93 (75.0)	44 (63.8)	0.10
Among Type-1 BAV patients	125 (84.5)	20 (76.9)	0.25
Among Type-2 BAV patients	2 (100.0)	2 (66.7)	0.60
Stroke/TIA within 30 days	6 (1.0)	3 (2.2)	0.21
30-day all-cause mortality	17 (2.7)	4 (2.9)	0.55

Values are *n* (%), median (IQR), or mean ± SD. * If multiple THVs were implanted, the size of the last implanted THV was recorded. BMI: body mass index; CT: computed tomography; LVEDD: left ventricular end-diastolic dimension; PVL: paravalvular leak; SOV: sinuses of Valsalva; STJ: sinotubular junction; STS: Society of Thoracic Surgeons; TIA: transient ischemic attack.

## Data Availability

The datasets are available upon request to the corresponding author.

## Supplementary Materials

The following supporting information can be downloaded at: https://www.mdpi.com/article/10.3390/jcm12155067/s1, Table S1: Baseline characteristics and procedural data in patients with first-generation and new-generation valves; Table S2: Baseline characteristics and procedural data (matched type-0 BAV subgroup); Table S3: Baseline characteristics and procedural data (matched type-1 BAV subgroup); Table S4: Baseline characteristics and procedural data (matched TAV subgroup); Table S5: Baseline characteristics and procedural data according to the approach of snare introduction; Moving image S1: The ipsilateral snare was pre-mounted on the delivery catheter just below the capsule; Moving image S2: The ipsilateral snare loop was unfastened and advanced to hold the initial third of the capsule; Moving image S3: The contralateral snare was placed in advance in the thoracic aorta and caught hold of the workhorse guidewire; Moving image S4: The contralateral snare held the initial third of the capsule; Moving image S5: Snare-assisted delivery of a self-expanding valve across the aortic valve improved the coaxiality of the system in horizontal aorta.

## Abbreviations

AA	annulus angulation
BAV	bicuspid aortic valve
BE	balloon-expandable
CT	computed tomography
LVEDD	left ventricular end-diastolic dimension
PSM	propensity score matching
PVL	paravalvular leak
SE	self-expanding
SOV	sinus of Valsalva
STJ	sinotubular junction
TAV	tricuspid aortic valve
TAVR	transcatheter aortic valve replacement

## References

[1] Leon M.B., Smith C.R., Mack M., Miller D.C., Moses J.W., Svensson L.G., Tuzcu E.M., Webb J.G., Fontana G.P., Makkar R.R. (2010). Transcatheter aortic-valve implantation for aortic stenosis in patients who cannot undergo surgery. N. Engl. J. Med..

[2] Leon M.B., Smith C.R., Mack M.J., Makkar R.R., Svensson L.G., Kodali S.K., Thourani V.H., Tuzcu E.M., Miller D.C., Herrmann H.C. (2016). Transcatheter or Surgical Aortic-Valve Replacement in Intermediate-Risk Patients. N. Engl. J. Med..

[3] Popma J.J., Deeb G.M., Yakubov S.J., Mumtaz M., Gada H., O’Hair D., Bajwa T., Heiser J.C., Merhi W., Kleiman N.S. (2019). Transcatheter Aortic-Valve Replacement with a Self-Expanding Valve in Low-Risk Patients. N. Engl. J. Med..

[4] Vincent F., Ternacle J., Denimal T., Shen M., Redfors B., Delhaye C., Simonato M., Debry N., Verdier B., Shahim B. (2021). Transcatheter Aortic Valve Replacement in Bicuspid Aortic Valve Stenosis. Circulation.

[5] Ochiai T., Yoon S.H., Sharma R., Miyasaka M., Maeno Y., Raschpichler M., Kashif M., Patel C., Patel V., Nomura T. (2020). Prevalence and Prognostic Impact of Ascending Aortic Dilatation in Patients Undergoing TAVR. JACC Cardiovasc. Imaging.

[6] Veulemans V., Maier O., Bosbach G., Polzin A., Piayda K., Afzal S., Jung C., Westenfeld R., Kelm M., Zeus T. (2020). Novel insights on outcome in horizontal aorta with self-expandable new-generation transcatheter aortic valve replacement devices. Catheter. Cardiovasc. Interv..

[7] Gallo F., Gallone G., Kim W.K., Reifart J., Veulemans V., Zeus T., Toggweiler S., De Backer O., Søndergaard L., Mangieri A. (2021). Horizontal Aorta in Transcatheter Self-Expanding Valves: Insights From the HORSE International Multicentre Registry. Circ. Cardiovasc. Interv..

[8] Gorla R., De Marco F., Garatti A., Bianchi G., Popolo Rubbio A., Acerbi E., Casenghi M., Spagnolo P., Brambilla N., Testa L. (2021). Impact of aortic angle on transcatheter aortic valve implantation outcome with Evolut-R, Portico, and Acurate-NEO. Catheter. Cardiovasc. Interv..

[9] Mangieri A., Tchetchè D., Kim W.-K., Pagnesi M., Sinning J.-M., Landes U., Kornowski R., Backer O.D., Nickenig G., Ielasi A. (2020). Balloon Versus Self-Expandable Valve for the Treatment of Bicuspid Aortic Valve Stenosis. Circ. Cardiovasc. Interv..

[10] Kim W.K., Blumenstein J., Liebetrau C., Rolf A., Gaede L., Van Linden A., Arsalan M., Doss M., Tijssen J.G.P., Hamm C.W. (2017). Comparison of outcomes using balloon-expandable versus self-expanding transcatheter prostheses according to the extent of aortic valve calcification. Clin. Res. Cardiol..

[11] Buono A., Medda M., Cesna S., Davidavicius G., Casilli F., Bande M., Pellicano M., Tespili M., Ielasi A. (2021). Snaring the Transcatheter Heart Valve Delivery System During Aortic Valve Replacement: When and Why. Cardiovasc. Revasc. Med..

[12] Espinoza Rueda M.A., Muratalla González R., García J.F., Morales Portano J.D., Alcántara Meléndez M.A., Jiménez Valverde A.S., Rivas Gálvez R.E., Campos Delgadillo J.L., González C.L., Gayosso Ortiz J.R. (2021). Description of the Step-by-Step Technique With Snare Catheter for TAVR in Horizontal Aorta. JACC Case Rep..

[13] Kaneko U., Hachinohe D., Kobayashi K., Fujita T. (2021). Snare-Assisted Valve Delivery to Overcome a Severely Calcified Aortic Arch during Transcatheter Aortic Valve Replacement. Korean Circ. J..

[14] Kunkel K.J., Fiorilli P., Kobayashi T., Desai N.D., Anwaruddin S., Herrmann H.C. (2021). Snare-Assisted Valve Positioning of Self-Expanding Valves for Transcatheter Aortic Valve Replacement. JACC Case Rep..

[15] Medda M., Casilli F., Tespili M., Bande M. (2020). The “Chaperone Technique”: Valve-in-Valve TAVR Procedure with a Self-Expandable Valve Inside a Degenerated Sutureless Prosthesis. JACC Cardiovasc. Interv..

[16] Marchese A., Tito A., Resta F., Tarantini G. (2021). Transcatheter Therapies in Challenging Aortic Ailments: TAVR in Extreme Horizontal Aortic Root With CoA Stenting. JACC Case Rep..

[17] Sievers H.H., Schmidtke C. (2007). A classification system for the bicuspid aortic valve from 304 surgical specimens. J. Thorac. Cardiovasc. Surg..

[18] Xiong T.Y., Li Y.J., Feng Y., Liao Y.B., Zhao Z.G., Mylotte D., Wei X., Xu Y.N., Peng Y., Wei J.F. (2019). Understanding the Interaction Between Transcatheter Aortic Valve Prostheses and Supra-Annular Structures From Post-Implant Stent Geometry. JACC Cardiovasc. Interv..

[19] Abramowitz Y., Maeno Y., Chakravarty T., Kazuno Y., Takahashi N., Kawamori H., Mangat G., Cheng W., Jilaihawi H., Makkar R.R. (2016). Aortic Angulation Attenuates Procedural Success following Self-Expandable but Not Balloon-Expandable TAVR. JACC Cardiovasc. Imaging.

[20] Di Stefano D., Colombo A., Mangieri A., Gallone G., Tzanis G., Laricchia A., Baldetti L., Palmisano A., Esposito A., Gallo F. (2019). Impact of horizontal aorta on procedural and clinical outcomes in second-generation transcatheter aortic valve implantation. EuroIntervention.

[21] D’Ancona G., Kische S., El-Mawardy M., Dißmann M., Heinze H., Zohlnhöfer-Momm D., Gürer H., Ince H. (2019). Aortic annulus angulation does not attenuate procedural success of transcatheter aortic valve replacement using a novel self-expanding bioprosthesis. Heart Vessel..

[22] Xiong T.Y., Ali W.B., Feng Y., Hayashida K., Jilaihawi H., Latib A., Lee M.K., Leon M.B., Makkar R.R., Modine T. (2022). Transcatheter aortic valve implantation in patients with bicuspid valve morphology: A roadmap towards standardization. Nat. Rev. Cardiol..

[23] Philip F., Faza N.N., Schoenhagen P., Desai M.Y., Tuzcu E.M., Svensson L.G., Kapadia S.R. (2015). Aortic annulus and root characteristics in severe aortic stenosis due to bicuspid aortic valve and tricuspid aortic valves: Implications for transcatheter aortic valve therapies. Catheter. Cardiovasc. Interv..

[24] Tchetche D., de Biase C., van Gils L., Parma R., Ochala A., Lefevre T., Hovasse T., De Backer O., Sondergaard L., Bleiziffer S. (2019). Bicuspid Aortic Valve Anatomy and Relationship With Devices: The BAVARD Multicenter Registry. Circ. Cardiovasc. Interv..

