# Supplementary material for: Snaring Self-Expanding Devices to Facilitate Transcatheter Aortic Valve Replacement in Patients with Complex Aortic Anatomies
Source: J Clin Med. 2023 Aug 1;12(15):5067. doi: 10.3390/jcm12155067 (PMC10420027; doi:10.3390/jcm12155067)
Supplement: Supplementary file 1 [file jcm-12-05067-s001.zip › Supplementary materials.pdf]

Moving image S1: The ipsilateral snare was pre-mounted on the delivery catheter just below the capsule.

Moving image S2: The ipsilateral snare loop was unfastened and advanced to hold the initial third of the capsule.

Moving image S3: The contralateral snare was placed in advance in the thoracic aorta and caught hold of the workhorse guidewire.

Moving image S4: The contralateral snare held the initial third of the capsule.

Moving image S5: Snare-assisted delivery of a self-expanding valve across the aortic valve improved the coaxiality of the system in horizontal aorta.

Table S1. Baseline characteristics and procedural data in patients with first-generation and new-generation valves.

	TAVR with first-generation valves			TAVR with new-generation valves		
**Variable**	**Non-snare group (N=433)**	**Snare group (N=73)**	**P**	**Non-snare group (N=194)**	**Snare group (N=66)**	**P**
Age, yrs	73±8	74±8	0.38	73±8	73±7	0.85
Male	242(55.9)	36(49.3)	0.30	110(56.7)	40(60.6)	0.58
BMI, kg/m^2	22.9±3.4	23.6±4.0	0.08	23.1±3.7	24.3±3.5	0.02
Height, m	1.59±0.08	1.59±0.09	0.93	1.59±0.08	1.60±0.09	0.78
Hypertension	193(44.6)	33(45.2)	0.92	77(39.7)	33(50)	0.14
Diabetes	91(21.0)	18(24.7)	0.49	38(19.6)	17(25.8)	0.29
Cerebrovascular disease	80(18.5)	16(21.9)	0.49	15(7.7)	4(6.1)	0.45
Coronary artery disease	83(19.2)	19(26.0)	0.18	44(22.7)	15(22.7)	0.99
Atrial fibrillation	65(15.0)	11(15.1)	0.99	26(13.4)	7(10.6)	0.56
STS score, %	3.13(2.06-5.11)	2.78(1.94-4.38)	0.40	2.23(1.58-3.32)	2.32(1.85-3.59)	0.57
Echocardiographic data						
Ejection fraction, %	56±15	59±14	0.15	57±16	60±13	0.24
Mean aortic valve gradient, mmHg	56±18	59±21	0.18	55±20	52±19	0.40
Peak aortic valve velocity, m/s	4.7±0.7	4.8±0.8	0.31	4.6±0.8	4.6±0.8	0.70
Aortic regurgitation (≥ moderate)	125(28.9)	9(12.3)	0.003	57(29.5)	13(19.7)	0.12
LVEDD, mm	53±9	49±8	<0.001	52±9	51±7	0.38
CT data						
Annulus angulation, °	52.0±9.5	61.7±10.4	<0.001	52.0±8.9	58.6±9.7	<0.001
Valve Type			<0.001			<0.001
TAV	242(55.9)	25(34.2)		111(57.2)	16(24.2)	
Type-0 BAV	87(20.1)	37(50.7)		37(19.1)	32(48.5)	
Type-1 BAV	102(23.6)	10(13.7)		46(23.7)	16(24.2)	
Type-2 BAV	2(0.5)	1(1.4)		0(0.0)	2(3.0)	
Annular area, mm^2	457.9±115.8	432.8±111.2	0.09	515.8±436.6	492.7±112.3	0.68
Annular perimeter, mm	76.7±9.4	74.5±9.2	0.06	81.7±50.4	79.7±9.1	0.75
Valve calcium volume, mm^3	447(198-796)	503(127-761)	0.55	350(166-764)	413(101-1009)	0.60
SOV perimeter, mm	108.4±13.5	109.1±14.4	0.70	110.1±13.9	114.5±13.2	0.03
STJ diameter, mm	30.0±4.2	32.0±4.8	<0.001	30.1±3.8	32.4±4.3	<0.001
Left coronary ostium height, mm	13.6±3.5	13.7±3.6	0.86	13.2±2.7	15.1±4.5	<0.001
Right coronary ostium height, mm	15.3±3.7	15.2±3.2	0.79	15±3.6	15.8±3.7	0.12
Maximal ascending aorta diameter, mm	39.6±5.3	42.5±7.9	<0.001	39.8±4.5	42.7±4.6	<0.001
Procedural Data and Clinical Outcome						
THV size more than 26 mm*	74(17.1)	11(15.1)	0.67	90(46.4)	26(39.4)	0.12
Post-dilation	223(51.5)	37(50.7)	0.90	80(41.2)	25(37.9)	0.63

Contrast volume, ml	302.3±84.9	304.6±86.9	0.84	314.3±79.3	308.8±65	0.62
Need for a second valve	36(8.3)	8(11.0)	0.46	8(4.1)	6(9.1)	0.11
Permanent pacemaker implantation	84(19.4)	16(21.9)	0.62	25(12.9)	12(18.2)	0.29
PVL ≥ mild	118(27.3)	16(21.9)	0.34	38(19.6)	16(24.2)	0.42
PVL ≥ moderate	2(0.5)	0(0.0)	0.73	2(1.0)	1(1.5)	0.59
Cardiac tamponade	1(0.2)	0(0.0)	0.86	1(0.5)	1(1.5)	0.45
Aortic dissection	4(0.9)	1(1.4)	0.54	0(0.0)	0(0.0)	-
Vascular complication			0.55			0.51
Minor	22(5.1)	6(8.2)		8(4.1)	2(3.0)	
Major	5(1.2)	1(1.4)		0(0.0)	0(0.0)	
Bleeding			0.63			0.67
Type 1	6(1.4)	0(0.0)		3(1.5)	2(3.0)	
Type 2	3(0.7)	0(0.0)		1(0.5)	0(0.0)	
Type 3	1(0.2)	0(0.0)		1(0.5)	1(1.5)	
Device success at 30 days						
Among TAV patients	202(83.5)	19(76.0)	0.25	95(85.6)	14(87.5)	0.60
Among Type-0 BAV patients	65(74.7)	23(62.2)	0.16	28(75.5)	21(65.6)	0.36
Among Type-1 BAV patients	84(82.4)	6(60.0)	0.11	41(89.1)	14(87.5)	0.59
Among Type-2 BAV patients	2(100.0)	1(100.0)	-	-	1(50.0)	-
Stroke/TIA within 30 days	2(0.5)	1(1.4)	0.37	4(2.1)	2(3.0)	0.48
30-day all-cause mortality	11(2.5)	1(1.4)	0.46	6(3.1)	3(4.5)	0.41

Values are n (%), median (IQR) or mean ± SD.

* If multiple THVs were implanted, the size of the last implanted THV was recorded.

BMI: body mass index; CT: computed tomography; LVEDD: left ventricular end-diastolic dimension; PVL: paravalvular leak; SOV: sinuses of Valsalva; STJ: sinotubular junction; STS: Society of Thoracic Surgeons; TIA: transient ischemic attack.

Table S2. Baseline characteristics and procedural data (matched type-0 BAV subgroup)

Variable	Non-Snare group (N=60)	Snare group (N=60)	P
Age, yrs	71±7	71±7	0.73
Male	29(48.3)	33(55.0)	0.47
BMI, kg/m^2	23.1±3.7	23.2±3.4	0.85
Height, m	1.59±0.07	1.60±0.08	0.40
Hypertension	23(38.3)	22(36.7)	0.85
Diabetes	9(15.0)	11(18.3)	0.62
Cerebrovascular disease	7(11.7)	7(11.7)	1.00
Coronary artery disease	8(13.3)	10(16.7)	0.61
Atrial fibrillation	6(10.0)	7(11.7)	0.77
STS score, %	2.37(1.64-4.61)	2.63(1.85-4.81)	0.66
**Echocardiographic data**			
Ejection fraction, %	56.4±13.5	58.5±14.6	0.41
Mean aortic valve gradient, mmHg	68.6±23.4	63.3±21	0.19
Peak aortic valve velocity, m/s	5.2±0.9	5±0.9	0.42
Aortic regurgitation ≥ moderate	5(8.3)	3(5.0)	0.36
LVEDD, mm	50±9	49±6	0.22
**CT data**			
Annulus angulation, °	55.0±8.4	56.9±10.7	0.28
Annular area, mm^2	478.3±128.1	442.2±114	0.11
Annular perimeter, mm	78.3±10.6	75.3±9.4	0.10
Valve calcium volume, mm^3	562(268-890)	538 (189-951)	0.84
SOV perimeter, mm	108.5±13	109.3±13.4	0.74
STJ diameter, mm	31.9±4.8	32.6±4.3	0.40
Left coronary ostium height, mm	15.7±3.6	15.8±4.0	0.82
Right coronary ostium height, mm	16.2±4.8	16±3.3	0.83
Maximal ascending aorta diameter, mm	43.6±4.9	43.7±4.4	0.96
**Procedural Data and Clinical Outcome**			
New-generation valve	19(31.7)	24(40.0)	0.34
THV size more than 26 mm*	14(23.3)	8(13.3)	0.16
Post-dilation	43(71.7)	40(66.7)	0.55
Contrast volume, ml	296±77	303±74	0.60
Need for a second valve	12(20.0)	8(13.3)	0.33

Permanent pacemaker implantation	11(18.3)	2(3.3)	0.01
PVL ≥ mild	19(31.7)	14(23.3)	0.28
PVL ≥ moderate	1(1.7)	1(1.7)	0.75
Cardiac tamponade	1(1.7)	1(1.7)	0.75
Aortic dissection	1(1.7)	1(1.7)	0.75
Vascular complication			0.26
Minor	3(5.0)	7(11.7)	
Major	1(1.7)	0(0.0)	
Bleeding			0.38
Type 1	1(1.7)	0(0.0)	
Type 2	2(3.3)	0(0.0)	
Type 3	1(1.7)	1(1.7)	
Device success at 30 days	39(65.0)	36(60.0)	0.57
Stroke/TIA within 30 days	0(0.0)	2(3.3)	0.25
30-day all-cause mortality	1(1.7)	1(1.7)	0.75

Values are n (%), median (IQR) or mean ± SD.

* If multiple THVs were implanted, the size of the last implanted THV was recorded.

BMI: body mass index; CT: computed tomography; LVEDD: left ventricular end-diastolic dimension; PVL: paravalvular leak; SOV: sinuses of Valsalva; STJ: sinotubular junction; STS: Society of Thoracic Surgeons; TIA: transient ischemic attack.

Table S3. Baseline characteristics and procedural data (matched type-1 BAV subgroup)

Variable	Non-Snare group (N=39)	Snare group (N=22)	P
Age, yrs	71.2±10.2	75±5.9	0.11
Male	27(69.2)	13(59.1)	0.42
BMI, kg/m^2	23.3±3.3	24±3.3	0.38
Height, m	1.60±0.08	1.60±0.10	0.83
Hypertension	13(33.3)	8(36.4)	0.81
Diabetes	8(20.5)	6(27.3)	0.55
Cerebrovascular disease	5(12.8)	6(27.3)	0.14
Coronary artery disease	10(25.6)	5(22.7)	0.80
Atrial fibrillation	3(7.7)	3(13.6)	0.37
STS score, %	2.40(1.39-3.23)	2.83(2.01-4.53)	0.17
**Echocardiographic data**			
Ejection fraction, %	55.2±15.6	60.7±13.3	0.17
Mean aortic valve gradient, mmHg	52.4±17.3	52±13.5	0.91
Peak aortic valve velocity, m/s	4.6±0.7	4.6±0.6	0.60
Aortic regurgitation ≥ moderate	10(25.6)	4(18.2)	0.51
LVEDD, mm	52.6±10.3	48.9±5.2	0.06
**CT data**			
Annulus angulation, °	58.6±7.7	60.2±8.3	0.44
Annular area, mm^2	505.7±113.2	507.3±118.1	0.96
Annular perimeter, mm	80.6±8.7	80.8±9.4	0.94
Valve calcium volume, mm^3	577(197-1110)	609(313-1707)	0.87
SOV perimeter, mm	111.4±17.2	112.6±13.9	0.79
STJ diameter, mm	30.5±4.6	31.2±4.5	0.54
Left coronary ostium height, mm	12.5±2.7	12.8±3.1	0.68
Right coronary ostium height, mm	14.9±3.6	14.3±4.1	0.62
Maximal ascending aorta diameter, mm	40.6±4.5	41.0±11.1	0.84
**Procedural Data and Clinical Outcome**			
New-generation valve	21(53.8)	14(63.6)	0.46
THV size more than 26 mm*	11(28.2)	8(36.4)	0.51
Post-dilation	27(69.2)	6(27.3)	0.002
Contrast volume, ml	309±78	293±52	0.40

Need for a second valve	6(15.4)	2(9.1)	0.39
Permanent pacemaker implantation	14(35.9)	7(31.8)	0.75
PVL ≥ mild	15(38.5)	8(36.4)	0.87
PVL ≥ moderate	0(0.0)	0(0.0)	-
Cardiac tamponade	0(0.0)	0(0.0)	-
Aortic dissection	0(0.0)	0(0.0)	-
Vascular complication			0.16
Minor	4(10.3)	0(0.0)	
Major	0(0.0)	0(0.0)	
Bleeding	0(0.0)	0(0.0)	-
Device success at 30 days	29(74.4)	15(68.2)	0.61
Stroke/TIA within 30 days	0(0.0)	1(4.5)	0.36
30-day all-cause mortality	1(2.6)	2(9.1)	0.29

Values are n (%), median (IQR) or mean ± SD.

* If multiple THVs were implanted, the size of the last implanted THV was recorded.

BMI: body mass index; CT: computed tomography; LVEDD: left ventricular end-diastolic dimension; PVL: paravalvular leak; SOV: sinuses of Valsalva; STJ: sinotubular junction; STS: Society of Thoracic Surgeons; TIA: transient ischemic attack.

Table S4. Baseline characteristics and procedural data (matched TAV subgroup)

Variable	Non-Snare group (N=77)	Snare group (N=39)	P
Age, yrs	75±7	77±7	0.19
Male	47(61.0)	19(48.7)	0.21
BMI, kg/m^2	24.0±3.2	24.6±4.7	0.47
Height, m	1.61±0.07	1.58±0.09	0.07
Hypertension	43(55.8)	27(69.2)	0.16
Diabetes	23(29.9)	14(35.9)	0.51
Cerebrovascular disease	13(16.9)	5(12.8)	0.57
Coronary artery disease	16(20.8)	12(30.8)	0.24
Atrial fibrillation	17(22.1)	5(12.8)	0.17
STS score, %	4.69(2.81-5.85)	3.47(2.89-5.04)	0.25
**Echocardiographic data**			
Ejection fraction, %	57±14	60±11	0.17
Mean aortic valve gradient, mmHg	52±15	47±15	0.11
Peak aortic valve velocity, m/s	4.6±0.7	4.3±0.7	0.11
Aortic regurgitation (≥ moderate)	35(45.5)	11(28.2)	0.07
LVEDD, mm	54±9	52±9	0.24
**CT data**			
Annulus angulation, °	56.7±6.7	63.0±8.1	<0.001
Annular area, mm^2	568.8±657.7	440.6±101.0	0.23
Annular perimeter, mm	77.1±11.0	75.3±8.6	0.38
Valve calcium volume, mm^3	360(151-585)	288(133-466)	0.20
SOV perimeter, mm	112.9±15.8	111.9±15.0	0.74
STJ diameter, mm	31.0±4.1	30.8±4.2	0.80
Left coronary ostium height, mm	12.9±2.8	12.3±3.3	0.24
Right coronary ostium height, mm	16.0±4.0	15.2±3.4	0.26
Maximal ascending aorta diameter, mm	39.9±4.7	40.6±5.6	0.46
**Procedural Data and Clinical Outcome**			
New-generation valve	26(33.8)	14(35.9)	0.82
THV size more than 26 mm*	34(44.2)	13(33.3)	0.26
Post-dilation	26(33.8)	8(20.5)	0.13
Contrast volume, ml	294±73	309±85	0.32
Need for a second valve	5(6.5)	6(15.4)	0.12

Permanent pacemaker implantation	24(31.2)	14(35.9)	0.61
PVL ≥ mild	12(15.6)	6(15.4)	0.98
PVL ≥ moderate	0(0.0)	0(0.0)	-
Cardiac tamponade	0(0.0)	0(0.0)	-
Aortic dissection	0(0.0)	0(0.0)	-
Vascular complication			0.37
Minor	2(2.6)	1(2.6)	
Major	0(0.0)	1(2.6)	
Bleeding			0.11
Type 1	0(0.0)	2(5.1)	
Type 2	0(0.0)	0(0.0)	
Type 3	0(0.0)	0(0.0)	
Device success at 30 days	67(87.0)	31(79.5)	0.29
Stroke/TIA within 30 days	0(0.0)	0(0.0)	-
30-day all-cause mortality	0(0.0)	0(0.0)	-

Values are n (%), median (IQR) or mean ± SD.

* If multiple THVs were implanted, the size of the last implanted THV was recorded.

BMI: body mass index; CT: computed tomography; LVEDD: left ventricular end-diastolic dimension; PVL: paravalvular leak; SOV: sinuses of Valsalva; STJ: sinotubular junction; STS: Society of Thoracic Surgeons; TIA: transient ischemic attack.

Table S5. Baseline characteristics and procedural data according to the approach of snare introduction.

Variable	Contralateral group (N=65)	Ipsilateral group (N=74)	P
Age, yrs	73±7	74±7	0.30
Male	36(55.4)	40(54.1)	0.88
BMI, kg/m^2	24.1±3.5	23.8±4.0	0.70
Height, m	1.59±0.09	1.59±0.09	0.98
Hypertension	29(44.6)	37(50.0)	0.53
Diabetes	11(16.9)	24(32.4)	0.04
Cerebrovascular disease	7(10.8)	13(17.6)	0.26
Coronary artery disease	12(18.5)	22(29.7)	0.12
Atrial fibrillation	7(10.8)	11(14.9)	0.47
STS score, %	2.33(1.89-3.57)	2.76(1.93-4.27)	0.17
**Echocardiographic data**			
Ejection fraction, %	58±14	60±13	0.60
Mean aortic valve gradient, mmHg	55±19	57±21	0.67
Peak aortic valve velocity, m/s	4.7±0.8	4.7±0.9	0.98
Aortic regurgitation (≥ moderate)	11(16.9)	11(14.9)	0.74
LVEDD, mm	51±7	49±8	0.21
**CT data**			
Annulus angulation, °	59.6±10.6	60.8±9.7	0.48
Valve Type			0.30
TAV	15(23.1)	26(35.1)	
Type-0 BAV	37(56.9)	32(43.2)	
Type-1 BAV	11(16.9)	15(20.3)	
Type-2 BAV	2(3.1)	1(1.4)	
Annular area, mm^2	471.8±115.3	449.9±115.0	0.27
Annular perimeter, mm	77.8±9.6	76.0±9.4	0.28
Valve calcium volume, mm^3	466(96-995)	438(138-720)	0.96
SOV perimeter, mm	112.7±13.2	110.6±14.8	0.40
STJ diameter, mm	32.6±4.6	31.8±4.5	0.32
Left coronary ostium height, mm	15.0±4.7	13.8±3.4	0.09
Right coronary ostium height, mm	15.8±3.3	15.2±3.5	0.30
Maximal ascending aorta diameter, mm	43.5±4.7	41.8±7.8	0.13

**Procedural Data and Clinical Outcome**			
New-generation valve	49(75.4)	17(23.0)	<0.001
THV size more than 26 mm*	19(29.2)	18(24.3)	0.51
Post-dilation	26(40.0)	36(48.6)	0.31
Contrast volume, ml	308±64	305±87	0.82
Need for a second valve	11(16.9)	8(10.8)	0.30
Permanent pacemaker implantation	11(16.9)	17(23.0)	0.38
PVL ≥ mild	21(32.3)	11(14.9)	0.01
PVL ≥ moderate	1(1.5)	0(0.0)	0.47
Cardiac tamponade	1(1.5)	0(0.0)	0.47
Aortic dissection	1(1.5)	0(0.0)	0.47
Vascular complication			0.43
Minor	5(7.7)	3(4.1)	
Major	0(0.0)	1(1.4)	
Bleeding			0.56
Type 1	1(1.5)	1(1.4)	
Type 2	0(0.0)	0(0.0)	
Type 3	1(1.5)	0(0.0)	
Device success at 30 days	41(63.1)	51(68.9)	0.47
Stroke/TIA within 30 days	1(1.5)	2(2.7)	0.55
30-day all-cause mortality	3(4.6)	1(1.4)	0.26

Values are n (%), median (IQR) or mean ± SD.

* If multiple THVs were implanted, the size of the last implanted THV was recorded.

BMI: body mass index; CT: computed tomography; LVEDD: left ventricular end-diastolic dimension; PVL: paravalvular leak; SOV: sinuses of Valsalva; STJ: sinotubular junction; STS: Society of Thoracic Surgeons; TIA: transient ischemic attack.
